# The efficacy and safety of transcranial direct current stimulation in patients with ADHD: a systematic review and meta-analysis

**DOI:** 10.3389/fpsyt.2026.1747588

**Published:** 2026-04-10

**Authors:** Jie Li, Xinyu Hou, Qiongli Fan, Li Chen

**Affiliations:** 1 Department of Pediatrics, Sichuan Provincial People's Hospital Affiliated to University of Electronic Science and Technology of China, Chengdu, Sichuan, China; 2 Growth, Development and Mental Health Center of Children and Adolescents, Chongqing Key Laboratory of Child Neurodevelopment and Cognitive Disorders, National Clinical Research Center for Child Health and Disorders, Children’s Hospital of Chongqing Medical University, Chongqing, China; 3 Growth, Development and Mental Health Center of Children and Adolescents, Ministry of Education Key Laboratory of Child Development and Disorders, National Clinical Research Center for Child Health and Disorders, Children’s Hospital of Chongqing Medical University, Chongqing, China; 4 Department of Pediatrics, Second Affiliated Hospital, Army Medical University, People’s Liberation Army, Chongqing, China

**Keywords:** ADHD, attention-deficit/hyperactivity disorder, efficacy, safety, transcranial direct current stimulation

## Abstract

**Objective:**

This meta-analysis evaluated the efficacy and safety of transcranial direct current stimulation (tDCS) for treating Attention-Deficit/Hyperactivity Disorder (ADHD).

**Methods:**

Following PRISMA guidelines, we analyzed 28 randomized controlled trials (RCTs) involving 1,864 participants. Outcomes encompassed core ADHD symptoms, hot and cold executive functions (EFs)—including inhibitory control, working memory, and cognitive flexibility—as well as safety profiles based on adverse events. A multilevel meta-analysis was performed using a random-effects model. Subgroup analyses and meta-regressions were conducted to explore potential moderating factors.

**Results:**

Compared to sham stimulation, tDCS did not significantly improve core ADHD symptoms (standardized mean difference (SMD) = –0.29, 95% CI [–0.59, 0.01], p= 0.05). Similarly, no significant overall effects were observed for cold EFs: inhibitory control (Hedges’ g(g)= –0.11, 95% CI [–0.26, 0.05], p=0.19), working memory (g= 0.13, 95% CI [–0.06, 0.32], p= 0.26), or cognitive flexibility (SMD = –0.42, 95% CI [–1.13, 0.29], p= 0.24). The effect on hot EFs was also non-significant (g = 0.27, 95% CI [–0.14, 0.70], p = 0.19). Exploratory analyses indicated that anode placement at Fp2 was associated with improvement in both inhibitory control (g= –0.52, 95% CI [–0.93, –0.11], p=0.01) and working memory (g = 0.72, 95% CI [0.22, 1.22], p = 0.004), although the overall test for interaction was not significant for inhibitory control (p= 0.19). The most common adverse reactions were mild and transient local skin symptoms, such as itching and redness (RR = 1.42, p=0.04).

**Conclusion:**

tDCS was well-tolerated but did not demonstrate significant overall efficacy for core ADHD symptoms or executive functions. Anodal stimulation at Fp2 showed potential selective benefits warranting further investigation. tDCS is not currently recommended as a standalone treatment for ADHD. Future research should optimize stimulation protocols and explore combined interventions with behavioral or cognitive therapies.

**Systematic Review Registration:**

https://www.crd.york.ac.uk/PROSPERO, identifier CRD42024612055.

## 1 Introduction

Attention-deficit/hyperactivity disorder (ADHD) is a common neurodevelopmental disorder that usually manifests before age 12 and can affect individuals throughout their lifespan. The global prevalence of ADHD is approximately 5%-7%, with an increasing prevalence in recent years (1). In the United States, the rate of diagnosis of ADHD among children was about 6%-8% in 2000, rising to approximately 11.4% by 2022, which means that some children in every classroom are likely to be affected by this disorder. ADHD not only affects the academic performance, social skills, and family relationships of affected children but also has long-term negative effects on their mental health, career development, and social adaptation in adulthood (2). The total economic burden on children with ADHD as they grow up is more than five times higher than that of typically developing children, placing a heavy financial strain on individuals, families, and society (3), thereby making it a public health issue that warrants significant attention.

ADHD, as a neurodevelopmental disorder, involves the delayed or abnormal development of the prefrontal cortex, which serves as the physiological basis for ADHD’s core symptoms (inattention, hyperactivity, and impulsivity) and is closely linked to deficits in Executive Function (EF) (4, 5).EF can be further divided into two distinct components—cold and hot—based on functional differences within prefrontal brain circuits (6). Specifically, cold EF involves cognitive control processes, such as Working Memory (WM), inhibitory control, and cognitive flexibility (7), whereas hot EF is associated with cognitive processing closely tied to emotion and motivation, including functions like delay of gratification, reward processing, and emotional regulation (8–10).EF deficits are the core cognitive deficits in children with ADHD and are highly correlated with their clinical core symptoms (9–11). Given that the prefrontal cortex undergoes rapid development during childhood, early intervention is critical to enhancing the brain’s plasticity during development, thereby improving EF (12). This improvement may contribute to alleviating the core cognitive deficits in children with ADHD.Therefore, early intervention for children with ADHD is critically important.

Traditional ADHD treatment methods primarily consist of medication and psychosocial and behavioral interventions (13). Medication, such as central nervous system stimulants and non-stimulants, can moderately improve symptoms. However, they come with several limitations. For example, medication can cause various side effects, such as insomnia, decreased appetite, and cardiovascular abnormalities (14), and some children may respond poorly to medication or develop tolerance, leading to reduced efficacy. In addition to medication, psychosocial and behavioral interventions can reduce behavioral problems and improve social skills in children with ADHD. These interventions often require a longer treatment duration and greater adherence, and their effectiveness can vary in real-world settings (15). Therefore, exploring new, more effective, and safer treatment methods is particularly important for ADHD patients.

Transcranial Direct Current Stimulation (tDCS) is a non-invasive brain stimulation method that has gained widespread attention in recent years in the field of neuroscience and is considered a promising neuromodulation technology. tDCS regulates neuronal excitability by applying weak direct current to the cerebral cortex, with benefits such as being easy to use, safe, and non-invasive to deeper brain tissues (16, 17). Multiple randomized controlled trials (RCTs) have reported positive effects of tDCS on cognitive functions (such as WM and inhibitory control) and clinical symptoms in ADHD patients (18, 19), but current research results still show some inconsistencies. Some studies have faced issues such as small sample sizes, insufficiently rigorous study designs, and differences in intervention protocols, which affect the credibility of their results. A meta-analysis observed that the effects of tDCS treatment on inhibitory control, processing speed, and attention deficits in ADHD patients were not significant (20). However, subsequent RCTs have shown positive results, particularly in the improvement of inhibitory control and WM after tDCS treatment for ADHD (21). A more recent meta-analysis also indicated significant improvements in inhibitory control, WM, and attention deficits with tDCS (22). A recently published RCT reported that tDCS stimulation of the left dorsolateral prefrontal cortex (L-DLPFC) significantly improved WM accuracy, while stimulation of the right medial prefrontal cortex significantly improved interference control (23). Another study also found no significant difference between the tDCS group and the placebo group in terms of Continuous Performance Test(CPT) error rates, N-back error rates, Stop-Signal Reaction Time (SSRT), and Stroop interference and facilitation. This indicates that tDCS does not have a significant effect on inhibition or interference control (24). Although tDCS is generally considered safe, some studies have reported adverse reactions such as scalp tingling and headaches (25). the meta-analysis by Yin et al (22) did not systematically summarize relevant adverse reaction data, making it impossible to provide comprehensive safety conclusions. This article summarizes adverse reaction data, including headaches and skin irritation, through a meta-analysis to provide a more comprehensive safety reference for clinicians and patients. It also includes new studies published after 2024 to comprehensively reassess the potential improvement effects of tDCS on ADHD symptoms, providing evidence-based support for clinical practice and reference for future research.

## 2 Materials and methods

### 2.1 Research protocol and registration

This study follows the PRISMA guidelines for systematic reviews and meta-analyses and has been registered in the International Prospective Register of Systematic Reviews (PROSPERO, registration number CRD42024612055).

### 2.2 Inclusion and exclusion criteria

#### 2.2.1 Inclusion criteria

(1) Studies exploring the efficacy and/or safety of noninvasive brain stimulation in patients with ADHD; (2) Randomized controlled trials, cluster randomized trials, and crossover trials; (3) The control group is a sham stimulation control group; (4) Participants must be diagnosed with ADHD, regardless of age, gender, or ethnicity; (5) Studies published in English.

#### 2.2.2 Exclusion criteria

(1) Subjects are not ADHD patients; (2) Studies not focused on noninvasive brain stimulation for treating ADHD; (3) Non-randomized studies, case reports, and case series; (4) Control groups that are improperly designed or not clearly defined; (5) Incomplete data or inability to obtain the full text; (6) Studies not using a sham stimulation control group.

### 2.3 Literature search strategy

In the databases of PubMed, Embase, Cochrane Library, web of science and ClinicalTrials.gov, from January 1990 to April 7, 2025, keywords such as “noninvasive brain stimulation” and “tDCS” (transcranial direct current stimulation) were used (see S1) to search for randomized controlled trials, including those with sham controls, limited to English-language literature. We also conducted a supplementary search by screening the reference lists of included studies, relevant systematic reviews, and meta-analyses to identify any additional eligible studies.

### 2.4 Study selection and data extraction process

The study selection began with one author (J-L) removing duplicates and screening titles and abstracts for relevance. A full-text review was then conducted by two reviewers (J-L and XY-H) who applied inclusion and exclusion criteria, with discrepancies resolved by a third author (L-C). The extracted data included authors, year, country, research background, study design, patient characteristics, intervention protocols, and outcome measures. For crossover trials, pre-crossover data were prioritized. Two authors independently extracted data, with disagreements resolved by a third reviewer (L-C). Additionally, means, standard deviations (SD), and sample sizes were collected for each group, and missing data were addressed by contacting study authors or through imputation methods.For crossover trials, data from the first intervention phase (pre-crossover) were preferentially extracted and analyzed to minimize potential carryover effects. For studies that only reported overall effects (i.e., combined outcomes from both intervention periods), the aggregated effect estimates as presented in the original reports were uniformly adopted to ensure consistency and completeness in data extraction. All data sources for crossover trials—whether first-phase or overall data—are clearly indicated in **Table 1**.

**Table 1:** Transcranial direct current stimulation studies.

Study identification and design		Population				Intervention						Outcomes	
Studies	Design	N	Age (M ± SD) year	Male(%)	Meds during trial/Pre-trial washout period.	Anode/Cathode	Target	mA	Session	Duration (min)	Timing	Cognitive and clinical	Side effect
Breitling, et al., 2020 (45)	Randomized Double blind Sham controlled Crossover	14	13.30 ± 1.90	86.67	naive	F8/contralateral supra-orbital area	Right IFG	1	1	20	Online	N-back task^a^	No specific adverse effects reported
Soff, et al., 2017 (78)	Randomized Double blind Sham controlled Crossover	15	14.2 ± 1.2	80	naive	F3/vertex	L- DLPFC	1	5	20	Online	QB-Test,Parents’ version of a German Adaptive Diagnostic Checklist for ADHD (FBB-ADHD)^a^	mild tingling and itching sensation
Klomjai, et al., 2022) (71)	Randomized Double blind Sham controlled Crossover	11	8.55 ± 0.65	90.9	yes/no	Fp2/F3	DLPFC	1.5	5	15	Ofline	Go/no-go task,Auditory continuous performance task (CPT)^b^	No specific adverse effects reported
Dubreuil-Vall, et al., 2021) (67)	Randomized Double blind Sham controlled Crossover	active:20 sham:20	37.53 ± 15.16	50	no/30d	F3 or F4/null	left/right DLPFC	2	1	30	Ofline	Flanker task-incongruent trials, Stop signal task^b^	No specific adverse effects reported
Allenby, et al., 2018 (25)	Randomized Double blind Sham controlled Crossover	37	Adults n/a	74.29	no/2w	F3/right-supra orbital area	L-DLPFC	2	3	20	Online	Stop-signal task,N-back task,CPT^b^	Tingling Itching Sensation etal
Breitling, et al., 2016 (64)	Randomized Single blind Sham controlled Crossover	Active:21 Sham: 21	13.30 ± 1.90	100	no/24h	F8/back of the head back of the head/F8	Right IFG	1	1	20	Online	Flanker task^a^	Skin sensations,Concentration ental
Jacoby and Lavidor 2018 (70)		20	22.75 ± 2.80	45	naive	F3 and F4/below the inion	DLPFC	1.8	1	20	Ofline	MOXO -CPT)^b^	No specific adverse effects reported
Soltaninejad, et al., 2019 (19)		20	16.1 ± 0.85	n/a	naive	F3/Fp2 (right suproobital);Fp2/F3	L-DLPFC	1.5	1	15	Online	Go/no-go, stroop^b^	No specific adverse effects reported
Salehinejad,et al., 2022 (76)		11	8.86 ± 1.80	50	no detail	F4 and F3/shoulder	Left and right DLPFCs,	1.5	1	15	Online	Go/no-go, N-back,Wisconsin Card Sorting Test(WCST)^a^	No specific adverse effects reported
Salehinejad, et al., 2020 (75)		17	9.53 ± 1.50	70.59	naive	P4/left shoulder	r-PPC	1	1	23	Online	Go/no-go task,stroop^,b^	Tingling Itching Sensation et.al
Nejati, et al., 2022 (74)		24	9.25 ± 1.53	66.67	naive	F4/left forearm	R-DLPFC	1	1	20	Offline	Go/no-go N-back^b^	No specific adverse effects reported
Nejati, et al., 2021 (80)		24	9.25 ± 1.53	63.63	naive	F3/F4	R-DLPFC	1	1	20	online	Go/No-Go,Flank^b^	
Breitling-Ziegler, et al., 2021 (65)	Randomized Double blind Sham controlled Parallel	Active: 9 Sham: 13	Active:13.22 ± 2.39 Sham: 13.54 ± 1.45	86.36	no/24h	F8/contralateral supra orbital area	right IFG	0.5/0.25	5	20	Online	Go/no-go, N-back,Flanker,FBB-DISYPS	Painful sensations, etal
Barham, et al., 2022 (21)		Active:11 Sham: 11	22 ± 2.77	31.8	naive	F4/F3	R-DlPFC	2	5	20	Ofline	Stroop,Digit span,CPT	No specific adverse effects reported
Sotnikova, A., et al.2017 (79)		Active:13 Sham: 13	14.21 ± 1.28	76.9	no/96h	F3/Cz.	L- DLPFC	1	1	20	Ofline	Go/no-go,N-back	tingling and itching sensation
Krauel, et al., 2025 (23)		Study A active:16 sham:19 Study B active:17 sham:17	13.3 years (IQR, 11.9-14.9 years)	78	no/2w	AF3+F3/TP7+OZ; F6+F8/AFZ+P7	L-DLPFC;right IFG	1	10	20	Ofline	N-back,flanker, The ADHD rating scale	Headache, nasopharyngitis et al
Westwood et al., 2022 (29)		Active+CT:10 Sham+CT: 13	Active:12.9 ± 1.9 Sham: 14.4 ± 1.9	100	no/24h	F8;/F1,	rIFC	1	20	15	Ofline	CPT,WSCT,ADHD Rating Scale–IV	
Cachoeira, et al., 2017 (18)		Active: 9 Sham: 8	Active:31 ± 6.17 Sham:33.75 ± 3.65	47.06	no/1month	F4/F3	R-DLPFC	2	5	20	Offline	Adult ADHD Self-Report Scale symptom(ASRS)	Tingling Itching Sensation etal
Cosmo,et al., 2015 (66)		Active:30 Sham:30	Active: 31.83 ± 11.55 Sham:.32.67 ± 10.37	Active:56.67 Sham:60.0	Yes/no	F3/F4	L-DLPFC	1	1	20	Ofline	Go/no-go task	No specific adverse effects reported
Nejati and Estaji 2024 (72)	Randomized Single blind Sham controlled Crossover	22	9.16 ± 1.63	63.6	no/24h	F3/Fp2;Fp2/F3	Left dlPFC/right vmPFC	1.5	3	20	Online	Emotional stroop^b^	Tingling,Burning Sensatio Pain tal
Nejati, et al., 2020 (31)	Randomized double-blinded sham controlled trial	Experiment 1active:15 Experiment 2 active:10	9.6 ± 2.3	80	naive	F3/F4 Fp2/F3 F3/Fp2	L-DLPF,r-OFC	1	1	15	Offline	Go/no-go, N-back, WCSTt,Stroop	No specific adverse effects reported
Leffa, et al., 2022 (28)	Randomized double-blind sham-controlled pilot study	Active:32 Sham:32	Active:38.2 ± 10.3 Sham:38.4 ± 9.1	53	no/30day	F3/F4	L- DLPFC	2	28	n/a	Offline	the Adult ADHD Self-report Scale version 1.1 (CASRS)	Tingling Itching Sensation etal
Schertz, et al., 2022) (77)		active+CT:13 sham+CT:14	10.83 ± 1.79	72	no/1M	F3/vertex	L-DLPFC	1	12	20	online	Cambridge Neuro psychological Test Automated Battery (CANTAB) Vanderbilt ADHD Parent Rating Scale (VADPRS),CBCL, Behavior Rating Inventory of EF, (BRIEF)	Dizziness headache,iching sensation etal
Estaji, et al., 2024 (68)	Randomized Single-blinded complete crossover design	active:24	9.16 ± 1.57	75	no/12h	dlPFC(F3)/vmPFC(Fp2);(vmPFC(Fp2)/dlPFC(F3)	DLPFC;vmPFC	2	1	20	oneline	Emotional Go//no-go,Emotional 1-back^b^	Tingling,confusion et al
Guimarães, et al., 2023) (69)	Randomized Triple blind Sham controlled Crossover	15	11.2 ± 3.0	66.67	naive	F3/contralateral supra-orbital area	L- DLPFC	2	5	30	Offline	Visual attention test,Digit span test SNAP-IV,CBCL,Wechsler Intelligence Scale for Children-WISC-V^b^	Headache,iching sensation etal
Beiman, et al., 2025 (24)	Randomized,single-blind, sham controlled, pretest-post test design	active:12 sham:15 control:30	23.04 ± 2.2	36.36	no/12h	F3/F4	L- DLPFC	2	1	25	Offline	CPT,N-back,Stop-Signal,Stroop, ASRS	No specific adverse effects reported
Nejati V2020 (33)	Randomized Single blind Sham controlled Crossover	20	8.60 ± 1.56	80.0	naive/1m	vmPFC(Fp2)/dlPFC(F3);dlPFC (F3)/vmPFC(Fp2)	vmPFC dlPFC	1	3	15	oneline	Balloon analogue risk-taking task (BART);Chocolate delay discounting task (CDDT)^b^	No specific adverse effects reported
Nejati V2024 (73)		23	10.087± 2.02	95.6	no/24h	F3/Fp2;Fp2/F3; F3/right shoulder, Fp2/left shoulder	Left dlPFC Right-vmPFC	1.5	5	15	oneline	BART;CDDT^b^	Pain,iching,et.al

CT, cognitive training; vmPFC, ventromedial prefrontal cortex; dlPFC, dorsolateral prefrontal cortex; OFC, orbitofrontal cortex; rIFG, right Inferior frontal gyrus; r-PPC, right posterior parietal cortex. a= denotes extraction of first-phase data;b=denotes extraction of overall data.

### 2.5 Data synthesis and analysis

To minimize variability in the data, we divided clinical symptom measures into two domains: inattention and hyperactivity/impulsivity. Cognitive outcome measures were categorized into cold EF and hot EF. Cold EF, which refers to cognitive processes involved in goal-directed behavior without emotional involvement, included three domains: inhibitory control, WM, and cognitive flexibility. Additionally, safety indicators were assessed.

To assess hyperactivity/impulsivity symptoms, we selected the following subscale scores: the hyperactive/impulsive subscale of the Adult ADHD Self-Report Scale (ASRS);ADHD rating scale; the hyperactivity subscale of the German ADHD Adaptive Diagnostic Scale (FBB-ADHD); the Vanderbilt ADHD Parent Rating Scale (VADPRS); the Conners Adult ADHD Rating Scale (CAARS); and the German DISYPS-II diagnostic system (FBB-DISYPS). Additionally, we included the inhibition subscale from the Behavior Rating Inventory of EF (BRIEF). Furthermore, to assess inattention symptoms, we used the attention deficit subscale of the ASRS;ADHD rating scale; the FBB-ADHD; the VADPRS; the Child Behavior Checklist (CBCL); the CAARS; and the FBB-DISYPS.

Measures of inhibitory control under the cold EF domain were categorized according to their core measurement properties to ensure conceptual and methodological consistency in the analyses (the complete study-wise coding scheme is provided in **Supplementary Table 1**).Accuracy/error-based measures reflect the frequency of inhibitory failures, with higher scores indicating worse performance. These included the number or percentage of commission errors or false alarms on No-go trials in the Go/No-go task; error rates on incongruent trials in Flanker; the number of commission errors in CPTs, Flanker tasks, or the MOXO-CPT;and the error response in the Stroop task.Speed-based measures reflect the speed or efficiency of the inhibitory process, with lower scores indicating better performance. These included The Stop Signal Reaction Time (SSRT) from the Stop-Signal Task or the Cambridge Neuropsychological Test Automated Battery (CANTAB), as well as reaction times to incongruent trials in Flanker or Stroop tasks.

To ensure consistent interpretation of effect sizes, a uniform coding rule was applied:a negative standardized mean difference (SMD) always indicates a beneficial effect of the intervention(i.e., improvement in inhibitory control).

Working memory was assessed using the number of correct responses or accuracy in the N-back task, accuracy on the Digit Span Backward test, accuracy on the backward condition of the Corsi Block-Tapping Task, and spatial working memory scores from CANTAB. Cognitive flexibility was measured by perseverative errors on the Wisconsin Card Sorting Test (WCST).

Regarding hot EF, which involves cognitive processes influenced by emotion and motivation, indicators included the number of successfully inflated balloons in the Balloon Analog Risk Task and the hyperbolic discount rate in the chocolate delay discounting task. The hyperbolic discount rate reflects the degree to which individuals devalue rewards as a function of delay, indicating impulsive decision-making tendencies.

Concerning safety, the included literature reported safety monitoring parameters. The data were divided into continuous variables and binary variables for statistical analysis.Additionally, we performed pre-specified subgroup analyses to examine the influence of key moderators, including study design, task type, stimulation site/target region, stimulation intensity, number of sessions, and stimulation duration. These analyses aimed to identify potential sources of heterogeneity and to generate more tailored insights for clinical application.For multi-arm trials (e.g., studies containing multiple active stimulation groups sharing a single sham control group), we followed the Cochrane Handbook guidelines and merged the active intervention groups where clinically and theoretically justified to avoid double-counting of control group data.

### 2.6 Risk of bias assessment

The risk of bias in the included randomized controlled trials (both parallel and crossover designs) was evaluated using the Cochrane Risk of Bias tool. Two reviewers independently assessed each study across five domains: selection bias, performance bias, detection bias, attrition bias, and other sources of bias. Each domain was rated as “low risk,” “high risk,” or “unclear risk.” Disagreements were resolved through discussion, with arbitration by a third reviewer (L−C) when consensus could not be reached.

The certainty of evidence for primary outcomes was evaluated using the Grading of Recommendations, Assessment, Development and Evaluation (GRADE) framework. Evidence was initially rated as high certainty based on the randomized trial design and was subsequently downgraded according to the following criteria: risk of bias, inconsistency, indirectness, imprecision, and publication bias. Two reviewers independently applied these criteria to predefined outcomes (e.g., inhibitory control, working memory, and skin adverse reactions). Discrepancies were resolved through discussion. Final certainty ratings (high, moderate, low, or very low), along with corresponding effect estimates and reasons for downgrading, are summarized in the Summary of Findings (SoF) table (**Supplementary Table 1**).

### 2.7 Robustness verification of results and detection of selective publication bias

This study assessed the robustness of meta-analysis findings through sensitivity analysis by excluding studies sequentially to reevaluate effect sizes. Results were deemed stable if effect size changes remained below 5% after any exclusion. If not, reversals in effect direction or statistical significance indicated potential heterogeneity or anomalies, necessitating further investigation.

To further ensure the robustness of the findings, the study also examined publication bias using a two-step verification process involving graphical and statistical methods. Initially, funnel plots from RevMan 5.4.1 were used for graphical screening, and effect size estimates were plotted against sample size for preliminary assessment. It is important to note that having fewer than ten studies may increase the risk of unreliable or misleading results. Subsequently, statistical testing with the metafor package in R evaluated funnel plot asymmetry through Egger’s test. This step helped assess whether findings might be influenced by publication bias, thereby providing a more reliable framework for result verification.

### 2.8 Statistical methods

A partitioned analytical approach was adopted to suit different data structures. For studies contributing only a single, independent effect size, conventional random-effects meta-analysis was performed using RevMan 5.4. To address the non-independence of multiple correlated effect sizes from the same study, a multilevel meta-analysis model was fitted in R (v4.3.3).The primary endpoint was the immediately post-treatment outcome. Effects are reported as standardized mean differences (SMD) with 95% confidence intervals (CI) for continuous data and as risk ratios (RR) with 95% CI for dichotomous data, pooled via random-effects models.Between-study heterogeneity was assessed using the I² statistic (I² < 50%: low; 50–75%: moderate; >75%: high). Sensitivity analysis employed the leave-one-out method. Potential publication bias was evaluated via funnel plots. Statistical significance was set at p < 0.05 (two-sided).

Exploratory subgroup analyses and meta-regression were conducted to investigate heterogeneity. Moderators included categorical (e.g., task type, stimulation site, study design, age group) and continuous variables (e.g., stimulation intensity, session number, duration). For categorical moderators, mixed-effects subgroup analyses were performed, with QM test p-values reported; for continuous moderators, meta-regression was applied. Analyses prioritized trials with larger samples.Adverse event effect sizes were calculated as SMD for continuous measures (e.g., severity) and RR for dichotomous outcomes (e.g., incidence).

## 3 Results

### 3.1 Selection of research literature

**Figure 1:**
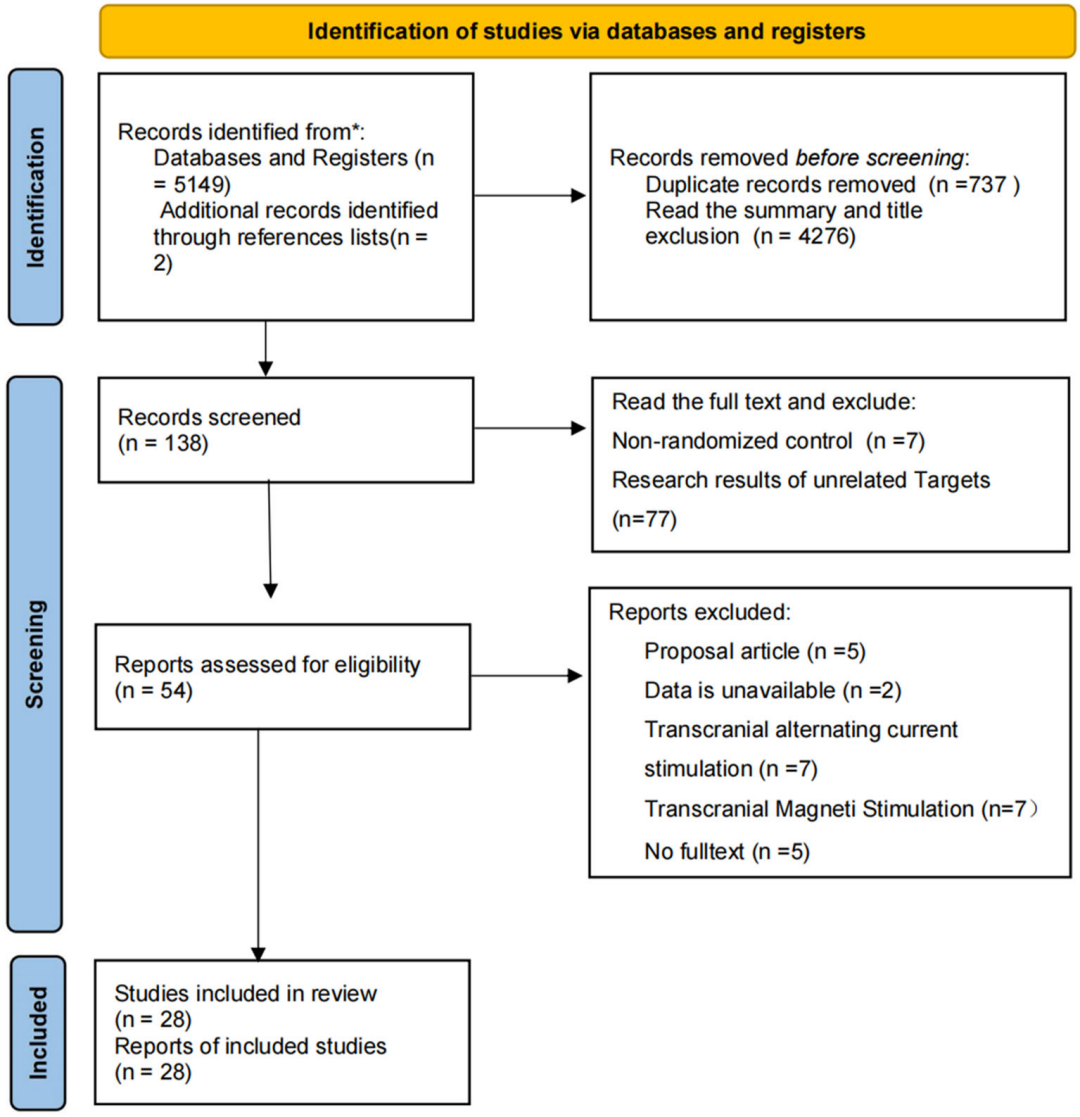
PRISMA flow diagram of the study selection process.

According to the PRISMA flowchart (**Figure 1**), our search strategy initially yielded 5,151citations: 368 from PubMed, 2,187 from EMBASE, 2,116 from the Cochrane Library clinical trial database, 18 from the ClinicalTrials database,458 from Web of science and 2 from reference. Additionally, two relevant references were identified through manual literature review. After removing 737 duplicate studies, a total of 4414 records remained. Following screening of titles and abstracts, we excluded 4,276 studies. From the remaining 138articles, we excluded 84 after reviewing the full texts for the following reasons: 7 non-randomized controlled studies; 77 studies and outcomes not relevant to the target condition. This left 54 articles for further scrutiny. We then excluded 26 of these for the following reasons: 5 full texts were unavailable; 5 were study protocols; 14 were not primary interventional studies; and 2 had data that were not available. Ultimately, 28 studies were included in the systematic review. **Figure 1** shows the selection process, and **Table 1** summarizes the general characteristics of the included studies.

### 3.2 Bias *risk ass*essment

**Figure 2:**
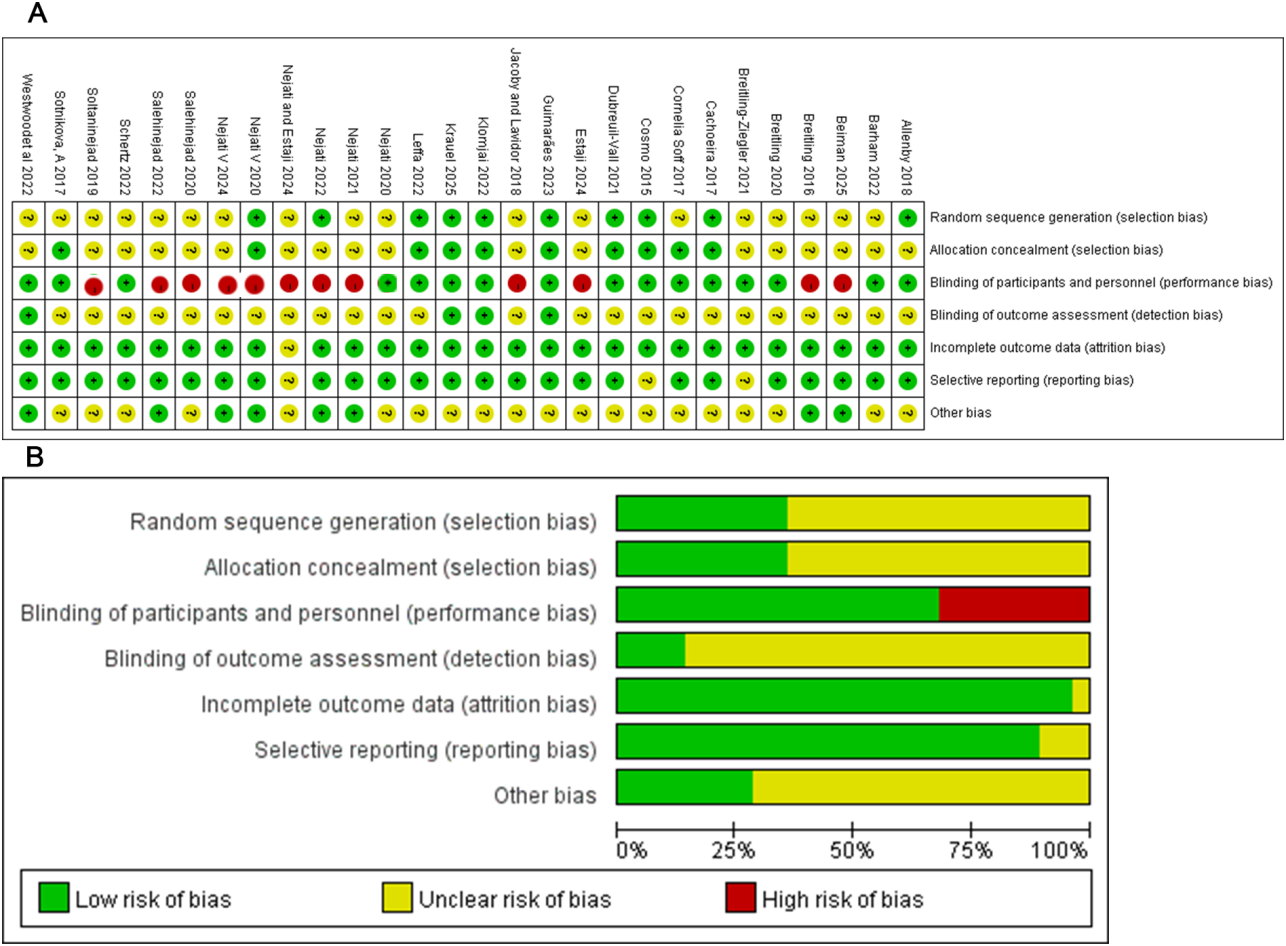
Risk of bias assessment for the included tDCS studies.​ **(A)** Risk of bias matrix. Each row represents an individual study, and each column represents a specific bias domain. Judgments are indicated with colored dots: green (low risk), yellow (unclear risk), and red (high risk). **(B)** Distribution of bias judgments. This stacked bar chart summarizes the percentage of studies rated as low, unclear, or high risk for each bias domain, highlighting the variation in methodological quality across domains.

The comprehensive assessment of bias risk for the target studies is presented in **Figure 2A** and **Figure 2B**. We evaluated 28randomized controlled trials, and the results are as follows. In “Random sequence generation (selection bias),” 18 studies did not specify the randomization method, and the risk of bias was unclear. In “Allocation concealment (selection bias),” 18 studies did not mention the concealment method, and the risk of bias was unclear. In “Blinding of participants and personnel (performance bias),” 12studies were single-blind, which indicates a higher risk of bias. In “Blinding of outcome assessment (detection bias),” 24studies had an unclear risk of bias. For “Incomplete outcome data (attrition bias)” and “Selective reporting (reporting bias),” the risk of bias was low across all studies. No high risk of bias was found in the category of “Other biases.”

### 3.3 tDCS *effect as*sessment

#### 3.3.1 Clinical *symptoms*

**Figure 3:**
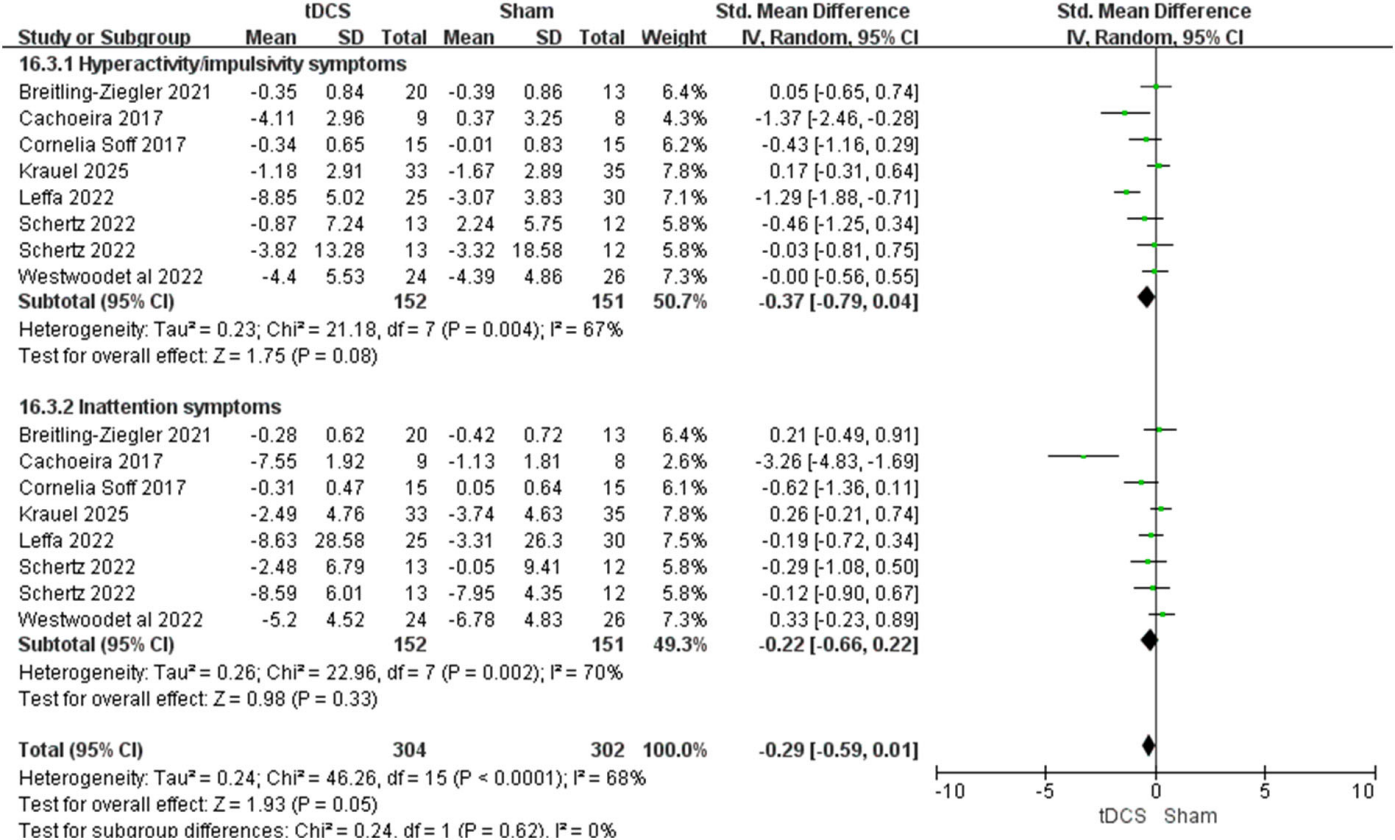
Forest plot of clinical symptoms.

A total of 7 studies involving 278 patients were included to evaluate the effects of tDCS on attention and hyperactive-impulsive behaviors in individuals with ADHD. The analysis showed no significant difference in clinical symptoms compared to the control group (**Figure 3** SMD = -0.29, 95% CI: -0.59 to 0.01, p = 0.05). In subgroup analyses, hyperactive-impulsive symptoms (SMD = -0.37, 95% CI: -0.79 to 0.04, p = 0.08) and attention deficit symptoms (SMD = -0.22, 95% CI: -0.66 to 0.22, p = 0.33) both showed no significant differences; however, both subgroups exhibited high statistical heterogeneity, with I² values of 67% and 70%, respectively. Further investigation revealed that the heterogeneity mainly originated from the studies by Cachoeira and Leffa, which included adult participants, whereas the other studies involved children. Excluding these two studies reduced heterogeneity to zero. Sensitivity analysis, excluding these two studies, did not alter the overall conclusion that tDCS did not significantly improve subjective clinical symptoms (attention and hyperactivity-impulsivity) in individuals with ADHD.

#### 3.3.2 Inhibition *control*

**Figure 4:**
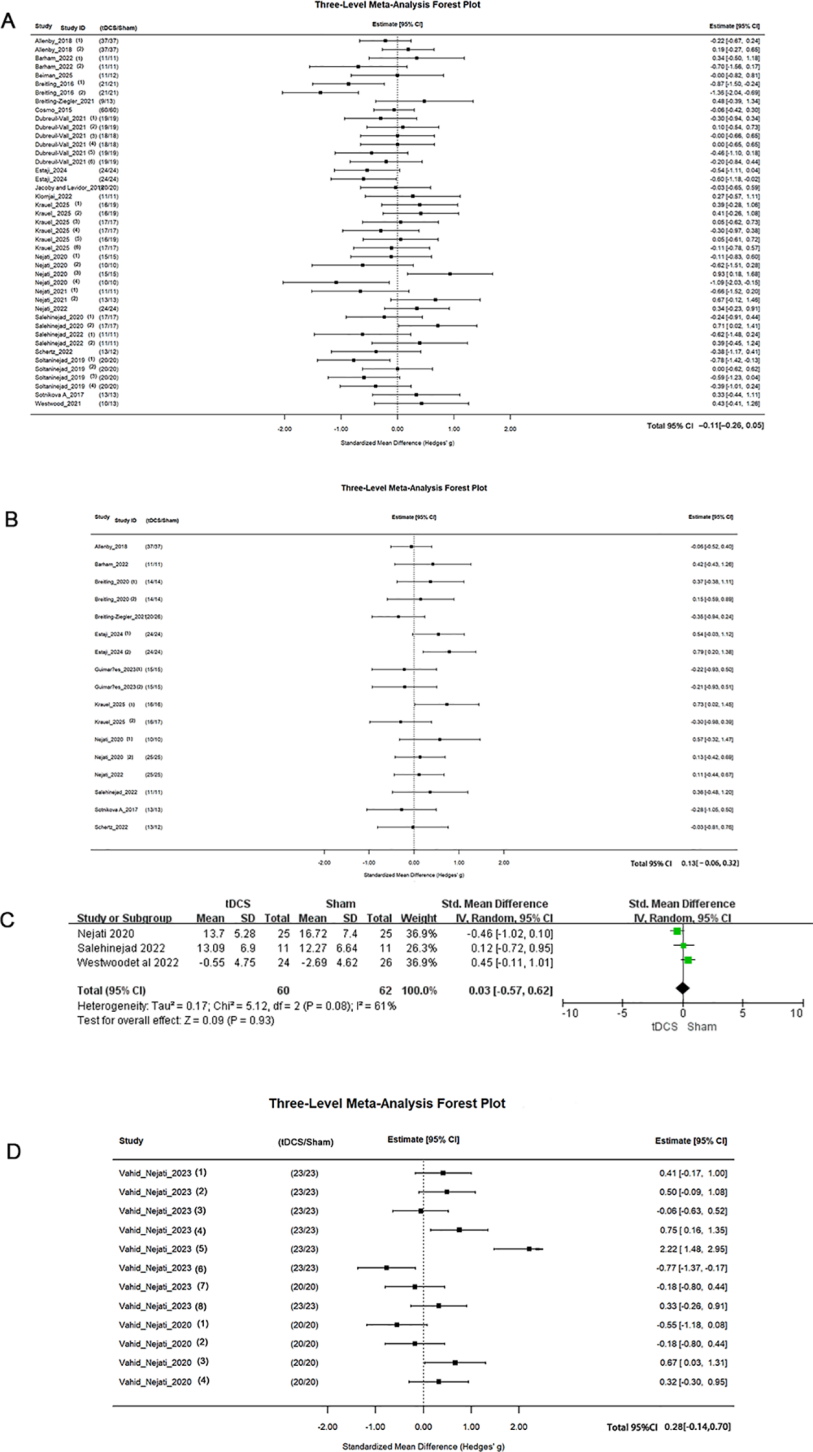
Forest plot of **(A)** inhibitory control, **(B)** work memory, **(C)** cognitive flexibility, **(D)** hot executive function in tDCS. The blue small squares represent the effect sizes of individual studies, and the black diamonds represent the group effect sizes.Each square represents the standardized mean difference (Hedges’ g) for a specific study, with the horizontal line indicating the 95% confidence interval (CI). The size of the square reflects the weight of the study in the analysis (18, 19, 21, 23–25, 28, 31, 33, 45, 64–79).

This three-level meta-analysis integrated 43 effect sizes from 18 tDCS studies (N = 872 children with ADHD) to evaluate tDCS effects on inhibitory control. The pooled effect was not statistically significant (**Figure 4A**, g(Hedges’ g)= –0.11, 95% CI [–0.26, 0.05], p = 0.19), with moderate heterogeneity (I² = 42.5%).

Exploratory moderator analyses revealed no significant effects for task type (CPT, Flanker, etc.; p = 0.55), study design (parallel vs. crossover; p =0.16), brain target (e.g. Dorsolateral Prefrontal Cortex (DLPFC)/Inferior Frontal Gyrus (IFG); p =0.60), or single-session duration (p = 0.51). Meta-regression showed a non-significant positive trend for the number of sessions (β = 0.03, p = 0.07) and no association with stimulation intensity (β = –0.17, p = 0.32).

Although anode location did not yield an overall significant difference (p =0.19), stimulation with the anode at Fp2 was associated with a significant improvement in inhibitory control (**Supplementary Table S2**,g = –0.52, 95% CI [–0.93, –0.11], p = 0.01). This finding is preliminary due to the non-significant overall test and limited subgroup samples, and requires further validation.

#### 3.3.3 Working *memory*

This three-level meta-analysis of 12 studies (17 effect sizes, N = 506 pediatric patients) evaluated the effect of tDCS on working memory. The overall pooled effect was not statistically significant (**Figures 4B,g** = 0.13, 95% CI [–0.06, 0.32], p = 0.26)with low heterogeneity (I² = 20.0%).

Exploratory moderator analyses found no significant effects for participant age (adult vs. pediatric; p = 0.44), brain target region (e.g., DLPFC, IFG; p = 0.69), stimulation duration (p = 0.50), or study design (parallel vs. crossover; p = 0.44). Meta-regression also showed no significant associations for stimulation intensity (β = 0.14, p =0.52) or number of sessions (β = –0.01, p = 0.65).

In contrast, subgroup analysis by electrode placement revealed a significant between-group difference (p = 0.04). Specifically, anode placement at Fp2 was associated with improved working memory (**Supplementary Table S3**,g = 0.72, 95% CI [0.22, 1.22], p = 0.004), whereas placements at F3, F4, or F8 showed no significant effect. Given the limited subgroup sample size, this finding should be considered a preliminary hypothesis requiring further validation in future research.

#### 3.3.4 Cognitive *flexibility*

We analyzed data from 3 studies with 122patients. Compared to the sham stimulation group, there was no significant difference in cognitive flexibility overall (**Figure 4C**, SMD=-0.42, 95% CI: [-1.13, 0.29], p=0.24).

#### 3.3.5 Hot *executive fun*ction

This three-level meta-analysis, which included 86 participants from two studies, evaluated the effect of tDCS on hot executive function. The pooled effect size did not reach statistical significance (**Figures 4D,g** = 0.27, 95% CI [–0.14, 0.70], p = 0.19). Overall heterogeneity was high (I² = 83%), and decomposition of heterogeneity indicated that most of the variance originated from within studies (within-study I² = 83%).

Meta-regression revealed no significant moderating effects for the number of stimulation sessions (**SupplementaryTable S4**,β = −0.16, p = 0.65) or stimulation intensity (β = −0.63, p = 0.64). Subgroup analysis by anode placement (e.g., F3, Fp2) also showed no statistically significant differences in effect sizes between locations (QM test, p = 0.39).

#### 3.3.6 Adverse *reactions*

**Figure 5:**
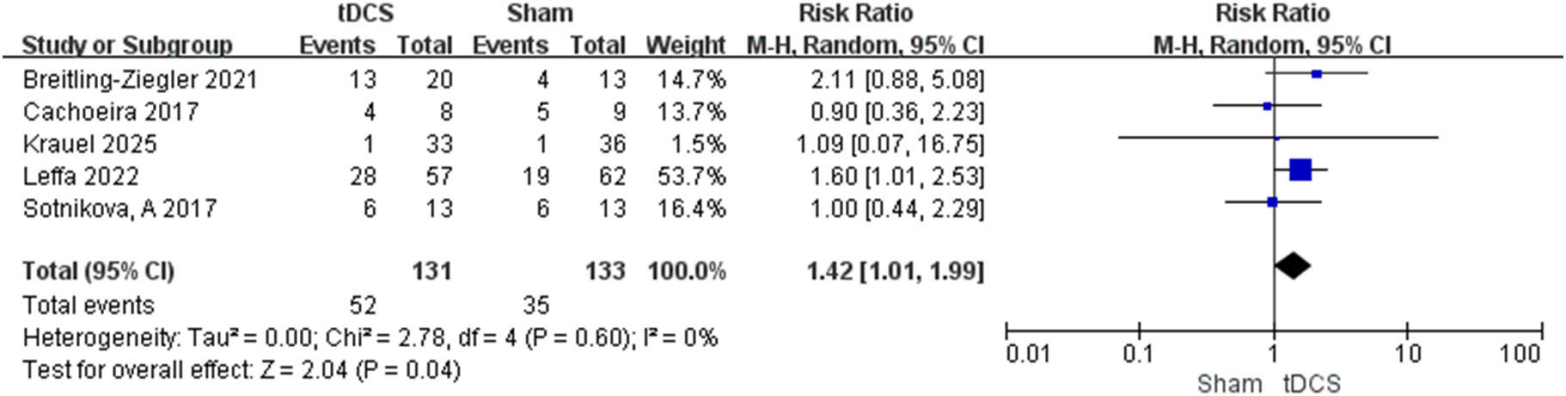
Forest plot of binary adverse skin-related symptoms in tDCS. Blue small squares represent the effect size of individual studies, and black diamonds represent the group effect size.(Cachoeira, Leffa et al., 2017, Soff, Sotnikova et al., 2017, Sotnikova, Soff et al., 2017, Breitling-Ziegler, Zaehle et al., 2021, Leffa, Grevet et al., 2022, Guimarães, Bandeira et al., 2023, Krauel, Brauer et al., 2025).

We analyzed adverse reactions by dividing the data into binary and continuous variables based on our recording method for separate analysis.For continuous data, there was no significant difference in adverse reactions between the treatment group and the sham stimulation group (**Supplementary Figure 1**, SMD = 0.07, 95% CI: [-0.09, 0.23], p=0.38). Analysis of binary data showed that skin-related symptoms (itching, local redness, etc.) occurred significantly more often in the treatment group than in the sham stimulation group (**Figure 5**, RR = 1.42, 95% CI: [1.01, 1.99], p=0.04), with no heterogeneity observed (I² = 0%). There were no significant differences between the two groups regarding neurological symptoms (see **Supplementary Figure 1**).

#### 3.3.7 Publication bias and sensitivity analysis

Since the number of available studies was limited, funnel plot analysis was conducted only for outcome indicators with data from more than 10 studies to assess publication bias. The results showed no significant evidence of publication bias for inhibitory control functions, as indicated by the funnel plot (**Supplementary Figure 2A**)and Egger’s test (β = 0.04, p=.98). For WM, Egger’s test (β = 0.96, *p* = 0.55) also did not detect significant asymmetry (**Supplementary Figure 2B**). These findings indicate that no significant publication bias was found in studies examining the effects of tDCS on cognitive functions (inhibitory control and WM). Sensitivity analysis demonstrated that. Sequential exclusion of each tDCS intervention study did not affect the results for cognitive functions (**Supplementary Table S5**).

## 4 Discussion

This study systematically reviewed and conducted a multi-level meta-analysis to comprehensively assess the effects of transcranial direct current stimulation (tDCS) on clinical symptoms, cold/hot executive functions, and safety in patients with ADHD. The main findings are summarized as follows: tDCS did not yield significant improvements in core clinical symptoms (inattention, hyperactivity/impulsivity) as assessed by rating scales; no significant overall effects were observed on cold executive functions, including inhibitory control, working memory, and cognitive flexibility; and the overall effect on hot executive functions was also non-significant. However, exploratory subgroup analyses suggested that a specific stimulation protocol with the anode positioned at Fp2 (approximating the right orbitofrontal cortex) may hold potential for enhancing inhibitory control and working memory. Regarding safety, tDCS was generally well-tolerated, but the incidence of skin-related adverse reactions (e.g., itching, local redness) was significantly higher compared to sham stimulation. The following sections discuss these findings comprehensively, interpret their implications in the context of methodological considerations, and compare and integrate them with conclusions from published systematic reviews.

### 4.1 Clinical symptoms

The present results indicate that active tDCS did not significantly improve the core clinical symptoms of ADHD relative to sham stimulation. This null finding aligns with the meta-analytic conclusion of Westwood et al. (2021) (20), who also reported no significant improvement in attention function based on purely objective cognitive tasks (e.g., CPT, Go/No-Go, Flanker; Hedges’ g= 0.18, p= 0.20). Together, these studies reveal the limited efficacy of current tDCS protocols for improving attention in ADHD from two distinct perspectives: subjective clinical symptoms and objective cognitive performance. However, our results contrast with meta-analyses by Yin et al. (22) and Zhang et al. (26), which found tDCS significantly improved inattention symptoms. This discrepancy likely stems from a combination of factors, including differences in the operationalization and measurement of “attention,” as well as heterogeneity in stimulation protocols.

First, essential differences exist in how studies operationalize the core construct of attention. This study focused on subjective clinical symptoms assessed via scales (e.g. ASRS, FBB-ADHD, VADPRS), reflecting functional impairments in daily life. In contrast, Yin et al. employed mixed indicators (22) combining scale scores and objective task performance, while Westwood et al. (20) and Zhang et al. (26) relied solely on objective cognitive paradigms (e.g., CPT, Go/No-Go). This measurement-level discrepancy means that the summarized “effects on attention” across these meta-analyses actually assess different constructs, rendering their results not directly comparable. Subjective symptom scales are susceptible to recall bias and social desirability effects, and patients in short-term interventions may struggle to perceive and accurately report subtle changes in daily functioning (26). Supporting this, Emser et al. (27)demonstrated a significant dissociation between self-reported symptoms and objective cognitive task performance in ADHD patients, suggesting these tools capture distinct dimensions of attentional function.

Second, methodological choices in meta-analysis, particularly the handling of multiple dependent effect sizes from the same study, can critically impact result stability. Ignoring the non-independence of effect sizes (e.g., treating multiple outcomes from one study as independent) may underestimate standard errors and inflate Type I error risk. Recent methodologies offer solutions; for instance, Westwood et al. (20) used a composite effect size approach based on assumed correlations, complemented by sensitivity analyses to enhance robustness. Zhang et al. (26)and Yin et al. (22) employed conventional random-effects models. These methodological differences may partially explain the inconsistent conclusions. Approaches that more directly model the nested data structure (e.g., multi-level models) can better partition within- and between-study variance, yielding more reliable parameter estimates and confidence intervals. Consequently, this study utilized a multi-level meta-analysis model (R metaforpackage), explicitly nesting effect sizes within studies. This method better accommodates the hierarchical data structure, helps control false-positive rates, and provides effect size estimates closer to the true population value.

Third, intervention duration may be inadequate. Meaningful improvement in clinical symptoms might require longer cumulative intervention. Leffa et al. (28) implemented the longest protocol to date—28 sessions over four weeks—and reported that benefits of right DLPFC stimulation on self-reported attention problems emerged only after 28 sessions, not after 14. Conversely, Westwood et al. (29) observed no clinical symptom improvement after 15 sessions of right inferior frontal cortex tDCS. Most studies included in our analysis had intervention cycles of 1–15 sessions, which may be insufficient to induce the neuroplastic changes necessary for clinical symptom improvement.

### 4.2 Cold executive function

The effects of tDCS on cold executive functions appear conditional and selective, showing both consistency with prior meta-analyses and novel insights.

We found the overall effect on inhibitory control was non-significant (g= -0.11, p= 0.19), with moderate heterogeneity (I² = 42.5%). This aligns with Westwood et al. (g= 0.21, p=0.06) (20). However, exploratory subgroup analyses revealed a notable pattern: the electrode configuration with the anode at Fp2 and cathode at the left frontal lobe (e.g., F3) was associated with a significant improvement in inhibitory control (g= -0.52, p= 0.01), whereas protocols with the anode at other sites (e.g., F3, F4) showed no significant effects. This finding has a neurobiological basis. The Fp2 region approximates the right orbitofrontal cortex (OFC)/ventromedial prefrontal cortex (vmPFC), which is crucial for response inhibition, impulse control, and reward-based decision-making (30). Applying cathodal stimulation to the left DLPFC (F3) may reduce inhibitory signaling to homologous right-hemisphere regions via the corpus callosum, thereby indirectly enhancing neural activity in the right DLPFC (19, 31).

Similarly, the overall effect on working memory was non-significant (g= 0.13, p= 0.26), with low heterogeneity (I² = 20.0%). The same Fp2-anode/F3-cathode configuration also showed a trend toward improvement (g= 0.72, p= .004). This pattern suggests this protocol may exert cross-domain regulatory effects on cold executive functions by modulating a broad neural network encompassing dorsal and ventral prefrontal cortices. While the dual-route model posits that the dorsal pathway (centered on the DLPFC) primarily mediates cold executive functions like working memory, and the ventral pathway (centered on the OFC/vmPFC) mediates affective and reward processing (32), evidence indicates considerable interaction. The DLPFC is involved in emotional processing (33–36), and the vmPFC contributes to executive processes including working memory (37), cognitive flexibility (38), and inhibitory control (38). These pathways interact closely in behavioral control; for instance, the DLPFC can modulate vmPFC value signals to facilitate self-control (39). Therefore, cathodal stimulation of the left DLPFC combined with anodal stimulation of the right OFC/vmPFC may produce cross-domain cognitive enhancement by modulating the balance between the dorsal control network and the ventral motivation-emotion network.

It is crucial to note that the findings from subgroup analyses are exploratory. The overall interaction tests did not achieve statistical significance for inhibitory control (p = 0.19) and reached only marginal significance for working memory (p = 0.04). Additionally, the number of studies within each subgroup was limited. Consequently, these results should be considered preliminary and hypothesis−generating rather than conclusive. Their validity and reproducibility require confirmation in future large−scale prospective studies. Furthermore, the present analysis did not identify significant moderating effects of task type, experimental design, stimulation intensity, or session number on either inhibitory control or working memory outcomes, indicating that these factors are unlikely to be major sources of heterogeneity in the existing evidence.

### 4.3 Hot executive function

The meta-analysis of hot executive functions showed a non-significant overall effect (g= 0.27, p=0.19), but with extremely high heterogeneity (I² = 83%), primarily from within-study sources. This result reflects a severe paucity of research on hot executive functions in this field. Hot executive functions involve cognitive processing of emotion and motivation, with neural substrates including the orbitofrontal cortex, ventromedial prefrontal cortex, anterior cingulate cortex, and ventral striatum (40). Notably, although the Fp2-anode configuration showed potential for cold executive functions, subgroup analyses for hot executive functions revealed no significant differences. The neural circuits underlying hot executive functions are more complex, involving dynamic interactions among multiple cortical and subcortical structures. A single stimulation protocol targeting the ventromedial prefrontal cortex may be insufficient to effectively modulate the entire network.

### 4.4 Safety

Our systematic safety analysis addresses a gap in previous meta-analyses. Unlike Yin et al. (2024) (22), who did not systematically summarize adverse events, this study analyzed adverse reactions as both continuous and binary outcomes. Results indicate tDCS was generally well-tolerated, with no significant difference in the severity of continuous adverse reactions compared to sham, consistent with the established safety profile of tDCS (41). However, the incidence of skin-related symptoms (itching, redness) was significantly higher in the active tDCS group (RR = 1.42,p=0.04), with no heterogeneity (I² = 0%). This finding has clinical relevance: although typically mild and transient, the increased incidence may be related to the physical properties of tDCS, including current delivery at the electrode-skin interface and electrolytic by-product deposition (42). The incidence of neurological (headache, dizziness) or other adverse reactions did not differ significantly between groups, further supporting the central nervous system safety of tDCS.

This encouraging safety conclusion should be interpreted cautiously, as adverse event reporting in the included studies was inconsistent and often incomplete. While existing evidence is valuable for clinical decision-making, potential underreporting may overestimate the certainty of conclusions and fail to fully reflect the true safety profile. In clinical practice, patients should be informed of potential skin reactions prior to treatment, and preventive measures should be implemented (e.g., adequate conductive gel application, electrode preparation, post-stimulation skin care). Future research should prioritize standardizing adverse event reporting and further explore the dose-response relationship between stimulation parameters (e.g., current intensity, electrode size, duration) and adverse reactions to optimize treatment safety.

### 4.5 Limitations of the intervention program

Traditional transcranial direct current stimulation (tDCS) typically uses bipolar electrodes measuring 7×5 cm in size, resulting in current dispersion and potentially causing the maximum current density to shift away from the target brain area (43). In contrast, high-definition transcranial direct current stimulation (HD-tDCS) can precisely focus the current using a 4×1 ring electrode configuration, significantly enhancing localization accuracy. It ensures effective stimulation of the target area, provides greater cortical current penetration, and minimizes unintended effects on non-target brain regions (43, 44). Studies have shown that HD-tDCS improves working memory (WM) in ADHD patients with effects correlated to individual symptom severity, with more significant positive effects in individuals with milder hyperactivity and impulsivity symptoms (45). Additionally, HD-tDCS can effectively modulate the activation state of the sensorimotor cortex in healthy subjects and improve various cognitive functions such as conflict control and WM (46, 47).

Given the complexity of ADHD neural network regulation, current tDCS research typically focuses on single-target stimulation—such as the dorsolateral prefrontal cortex (DLPFC), right inferior frontal gyrus (rIFG), or ventromedial prefrontal cortex (vmPFC)—while often neglecting the dynamic interactions between neural networks (48). The development of HD-tDCS offers new possibilities for targeted treatment. Future research could explore how multi-channel HD-tDCS influences network regulation by using resting-state functional magnetic resonance imaging (fMRI) to map abnormal brain regions—those showing atypical activity or connectivity in ADHD—that are functionally connected. This approach would enable more accurate and individualized neural modulation.

The core symptoms of ADHD exhibit high heterogeneity (49, 50), involving dysfunction of multiple distributed brain networks (Fischer, Fried et al., 2017, particularly the cingulate-prefrontal-parietal (CFP) network, as well as the functional connectivity between the dorsal anterior cingulate cortex (dACC) and the dorsolateral prefrontal cortex (DLPFC) (51). Current tDCS studies often adopt standardized stimulation protocols targeting single brain regions (such as DLPFC, rIFG, vmPFC), and this focal intervention may struggle to match the network-level complexity of ADHD, thereby limiting its effectiveness in improving diverse clinical symptoms. Notably, in addition to traditional targets, non-higher-order networks such as the sensorimotor network (SMN) may also become potential intervention targets for tDCS (52). The study by Kebets et al. (2019) (52) indicates that abnormal functional connectivity of the SMN has cross-diagnostic significance in various mental disorders, including ADHD, suggesting it may become a new target for tDCS intervention.

Animal experiments (53) and human studies (54) have confirmed that tDCS does not directly “create” memories or skills, but rather enhances experience-dependent synaptic plasticity that occurs synchronously with behavioral training. The animal experiments by Fritsch et al. (2010) (53) found that tDCS can only enhance the expression of synaptic plasticity markers (such as brain-derived neurotrophic factor, BDNF) and motor learning effects when applied synchronously with motor training. The human study by Reis et al. (54) also confirmed that when tDCS is applied alone without synchronous motor skill training, no learning benefits were observed. This finding suggests that tDCS may be more suitable as an adjunctive means of combined intervention rather than an independent treatment method. Future research should prioritize exploring synergistic strategies that combine tDCS with cognitive training, behavioral therapy, or pharmacotherapy.

### 4.6 Study limitations

Several limitations of this study should be acknowledged. First, although the meta-analysis incorporated 28 tDCS studies, substantial heterogeneity existed in stimulation protocols, particularly regarding electrode montages (e.g., unilateral vs. bilateral DLPFC stimulation, targeting the right inferior frontal gyrus vs. other sites). This variability may contribute to inconsistent outcomes across studies.Second, protocols for managing concomitant medications and washout periods varied across the included crossover trials, which may have introduced heterogeneity. Although we included “study design” as a moderator in subgroup analyses, this approach could not fully account for these unmeasured methodological differences.Third, the number of studies available for each specific subgroup analysis was limited, which increases the risk of false-positive findings. Consequently, these exploratory results should be interpreted as hypothesis-generating rather than definitive.Fourth, with the exception of three studies that enrolled more than 30 participants, the remaining trials were relatively small (average sample size ~15, range 7–24). This may limit statistical power and affect the generalizability of the pooled estimates.Furthermore, the meta-analysis was challenged by variability in outcome reporting. Some studies reported change scores (post-treatment minus baseline) to control for baseline differences, while others reported post-treatment scores only. This inconsistency complicates the synthesis of effect sizes.Finally, most included studies featured short follow-up periods (ranging from 1 to 7 days), which precludes assessment of the long-term durability of tDCS effects and their far transfer to daily functioning in individuals with ADHD.

### 4.7 Future research directions

Based on current evidence, tDCS is not yet suitable as a stand-alone treatment for the core symptoms of ADHD. The observed cognitive-enhancing effects are modest and require further investigation. Nevertheless, tDCS remains a promising adjunctive intervention with the potential to be combined with cognitive training, behavioral therapy, or pharmacotherapy to target specific cognitive deficits in ADHD. Future research should prioritize the following directions to advance the field.

First, a paradigm shift from focal stimulation to network-based modulation is essential for achieving precise targeting.Given the complexity of neural circuits implicated in ADHD, future studies should move beyond the traditional single-target approach (e.g., DLPFC) to include other ADHD-relevant regions, such as the right inferior frontal gyrus (rIFG) and the ventromedial prefrontal cortex (vmPFC). Crucially, the focus should transition from “single-target stimulation” to “multi-target network modulation.” Specific strategies include: (1) utilizing resting-state fMRI or EEG functional connectivity to identify patient-specific dysfunctional networks (e.g., the dACC-DLPFC network) (55), combined with high-definition tDCS (HD-tDCS) to enable precise “network-targeted” neuromodulation; and (2) optimizing multi-electrode tDCS (mtDCS) montages to achieve network-level regulation. For instance, Mencarelli et al. (56) demonstrated that placing the anode over the central executive network and the cathode over the default mode network (DMN) can concurrently enhance task-positive network activity and suppress task-negative network activity. Such network-level intervention strategies may better align with the multisystem dysregulation model of ADHD pathophysiology.

Second, stimulation parameters and combined intervention strategies require systematic optimization.​ The exploratory findings of this study indicate that the anode-Fp2/cathode-F3 montage may hold potential, a hypothesis that urgently requires validation in prospective, adequately powered trials. Concurrently, a unified standard for reporting stimulation parameters must be established. Studies should clearly report current density (or electric field strength) rather than merely current amplitude, especially when different electrode sizes are used. Considering that target regions like the IFG are situated at greater cortical depth, effective stimulation may necessitate higher current intensities to reach therapeutic thresholds (57). Furthermore, stimulation protocols—including intensity, session frequency, and overall duration—should be optimized. Given the cumulative nature of neuromodulatory effects, multi-session regimens over extended periods (e.g., four weeks) are recommended to achieve more sustained benefits. To address the clinical heterogeneity of ADHD, personalized treatment protocols based on individual symptom profiles and neurobiological characteristics should be developed. Combining tDCS with cognitive training or behavioral interventions could further enhance overall efficacy through multimodal synergy.

Third, integrating emerging technologies and interdisciplinary approaches is a promising avenue.​ Novel neuromodulation techniques, such as transcranial random noise stimulation (tRNS) (58) and transcranial alternating current stimulation (tACS) (59), partially overcome the limitations of conventional tDCS in reaching deeper brain regions and show preliminary potential for improving cognitive deficits and core symptoms in ADHD (60). However, research on tACS and tRNS for ADHD treatment is still in its infancy, warranting more rigorous investigation. Additionally, machine learning algorithms applied to neuroimaging data have been used to develop diagnostic classifiers for ADHD (61, 62). Future research should aim to integrate machine learning with multimodal data (symptoms, neuroimaging) to predict individual treatment responses—for example, identifying which patients are more likely to benefit from DLPFC versus vmPFC stimulation—thus paving the way for personalized non-pharmacological interventions (63).

Finally, while tDCS is generally safe with adverse events primarily limited to transient skin reactions, establishing a standardized monitoring and reporting system for adverse events is crucial to optimally balance efficacy and safety. Future studies should systematically document the incidence, severity, and duration of all adverse effects to better define the risk-benefit profile of tDCS protocols in ADHD.

## Data Availability

The original contributions presented in the study are included in the article/**Supplementary Material**. Further inquiries can be directed to the corresponding author.
