# Supplementary material for: The efficacy and safety of transcranial direct current stimulation in patients with ADHD: a systematic review and meta-analysis
Source: Front Psychiatry. 2026 Apr 10;17:1747588. doi: 10.3389/fpsyt.2026.1747588 (PMC13106418; doi:10.3389/fpsyt.2026.1747588)
Supplement: Supplementary file 1 [file Supplementaryfile1.docx]

Supplementary Material

**1,Supplementary 1(S1),Literature Search Strategy**

**Pubmed**

Search:((((((((("Attention Deficit and Disruptive Behavior Disorders"[Mesh]) OR "Attention Deficit Disorder with Hyperactivity"[Mesh])) OR "Conduct Disorder"[Mesh]) OR "Hyperkinesis"[Mesh]) OR ((ADHD[Title/Abstract] OR ADDH[Title/Abstract] OR ADHS[Title/Abstract] OR HKD[Title/Abstract] OR TDAH OR"AD/HD"[Title/Abstract]))) OR ((impulsiv*[Title/Abstract] OR inattentiv*[Title/Abstract] OR inattention*[Title/Abstract]))) OR ((hyperkin*[Title/Abstract] OR hyperactiv* [Title/Abstract]))) AND (((((("Transcutaneous Electric Nerve Stimulation"[Mesh]) OR "Transcranial Direct Current Stimulation"[Mesh]) OR "Electrodes"[Mesh]) OR "Electric Stimulation Therapy"[Mesh]) OR "Electric Stimulation"[Mesh]) OR ((((((((((transcranial[Title/Abstract]) OR (trans-cranial[Title/Abstract])) OR (noninvasive brain stimulation[Title/Abstract])) OR (tDCS[Title/Abstract])) OR (A-tDCS[Title/Abstract])) OR (C-tDCS[Title/Abstract])) OR (S-tDCS[Title/Abstract])) OR (electrode*[Title/Abstract])) OR (anod*[Title/Abstract])) OR (cathod*[Title/Abstract])))) AND (("randomized controlled trial"[Publication Type] OR "controlled clinical trial"[Publication Type] OR "randomized"[Title/Abstract] OR "placebo"[Title/Abstract] OR "randomly"[Title/Abstract] OR "trial"[Title/Abstract]) NOT ("animals"[Mesh] NOT "humans"[Mesh])) Filters:from 1990/1/1 - 2025/4/7

**Cochrane Library**

#1 MeSH descriptor: [Attention Deficit Disorder with Hyperactivity] explode all trees

#2 MeSH descriptor: [Conduct Disorder] explode all trees

#3 ((ADHD OR ADDH OR ADHS OR AD NEXT HD OR HKD OR TDAH)):ti,ab,kw (Word variations have been searched)

#4 (((attention* OR behav*) near/3 (defic* OR dysfunc* OR disorder*))):ti,ab,kw (Word variations have been searched)

#5 (((disrupt* NEAR/3 disorder*) OR (disrupt* NEAR/3 behav*) OR (defian*NEAR/3 disorder*) OR (defian* NEAR/3 behav*))):ti,ab,kw (Word variations have been searched)

#6 ((impulsiv* OR inattentiv* OR inattention*)):ti,ab,kw (Word variations have been searched)

#7 MeSH descriptor: [Hyperkinesis] explode all trees

#8 ((hyperkin* OR hyper NEXT kin*)):ti,ab,kw (Word variations have been searched)

#9 ((minimal* NEAR/3 brain NEAR/3 (disorder* OR dysfunct* OR damage*))):ti,ab,kw (Word variations have been searched)

#10 ((hyperactiv* OR hyper NEXT activ*)):ti,ab,kw (Word variations have been searched)

#11 {or #1-#10}

#12 MeSH descriptor: [Transcranial Direct Current Stimulation] explode all trees

#13 (TMS):ti,ab,kw OR (transcranial*):ti,ab,kw OR ("trans-cranial"):ti,ab,kw OR (magn*):ti,ab,kw (Word variations have been searched)

#14 MeSH descriptor: [Electric Stimulation Therapy] explode all trees

#15 MeSH descriptor: [Electric Stimulation] explode all trees

#16 (rTMS):ti,ab,kw OR (“Repetitive transcranial magnetic stimulation ”):ti,ab,kw OR ("brain stimulation "):ti,ab,kw (Word variations have been searched)

#17 MeSH descriptor: [Electrodes] explode all trees

#18 (transcranial):ti,ab,kw AND (“direct current ”):ti,ab,kw AND (stimulation):ti,ab,kw (Word variations have been searched)

#19 (transcranial):ti,ab,kw AND (DC):ti,ab,kw AND (stimulation):ti,ab,kw (Word variations have been searched)

#20 (transcranial):ti,ab,kw AND (electric*):ti,ab,kw AND (stimulation):ti,ab,kw (Word variations have been searched)

#21 (( electrode* OR anod* OR cathod* )):ti,ab,kw (Word variations have been searched)

#22 (tDCS):ti,ab,kw OR ("A-tDCS "):ti,ab,kw OR ("C-tDCS"):ti,ab,kw OR (" S-tDCS"):ti,ab,kw (Word variations have been searched)

#23 {or #12-#22}

#24 #11 AND #23

**Web of Science**

(TS=(Attention-deficit/hyperactivity disorder) OR TS=(ADHD) OR TS=(Attention Deficit Hyperactivity Disorder) OR TS=(Hyperkinetic Disorder) OR TS=(Attention Deficit Disorder) OR TS=(Hyperactivity Disorder)) AND (TS=(tDCS) OR TS=(Transcranial Direct Current Stimulation) OR TS=(Cranial Electrotherapy Stimulation) OR TS=(Direct Current Stimulation) OR TS=(Transcranial Stimulation))

**EMBASE**

#1 adhd:ab,ti OR 'attention adj3 deficit$':ab,ti OR addh:ab,ti OR adhs:ab,ti OR hyperactiv$:ab,ti

#2 'crossover procedure'/exp OR 'crossover procedure' OR 'double blind procedure' OR 'randomized controlled trial' OR 'single blind procedure'

#3 'attention deficit hyperactivity disorder'/exp

#4 #1 OR #3

#5 random*

#6 factorial* OR crossover* OR placebo* OR doubl* OR assign*

#7 allocat* OR volunteer* OR 'cross next/1 over*' OR 'doubl* near/1 blind*' OR 'singl* near/1 blind*'

#8 #2 OR #5 OR #6 OR #7

#9 'transcranial magnetic stimulation'/exp

#10 'transcranial magnetic stimulation'/exp OR 'transcranial direct current stimulation'/exp OR 'electrostimulation'/exp

#11 'transcranial magnetic stimulation'/exp OR 'transcranial magnetic stimulation' OR 'trans-cranial*' OR magnetic* OR tdcs OR transcranial*

#12 transcranial AND ('direct current'/exp OR 'direct current') AND ('stimulation'/exp OR stimulation)

#13 transcranial AND 'direct current':ab,ti AND stimulation:ab,ti

#14 transcranial AND dc:ab,ti AND stimulation:ab,ti

#15 transcranial AND electric*:ab,ti AND stimulation:ab,ti

#16 tdcs OR electrode*:ab,ti OR anode:ab,ti OR anodes:ab,ti OR anodal:ab,ti OR cathod*:ab,ti

#17 #9 OR #10 OR #11 OR #12 OR #13 OR #14 OR #15 OR #16

#18 #4 AND #8 AND #17

**ClinicalTrials.gov**

Condition or disease :ADHD

Other terms:'Transcranial Direct Current Stimulation'

# Supplementary Figures and Tables

## Supplementary Figures


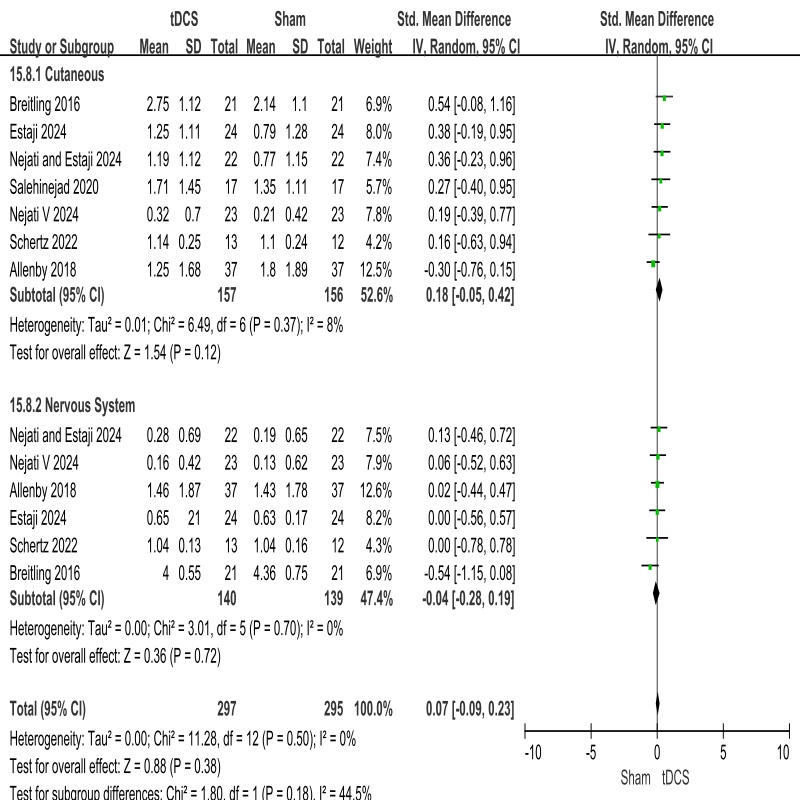


**Supplementar**y **Figure** S1.Forest plot of continuous variables for adverse effects in tDCS therapy.


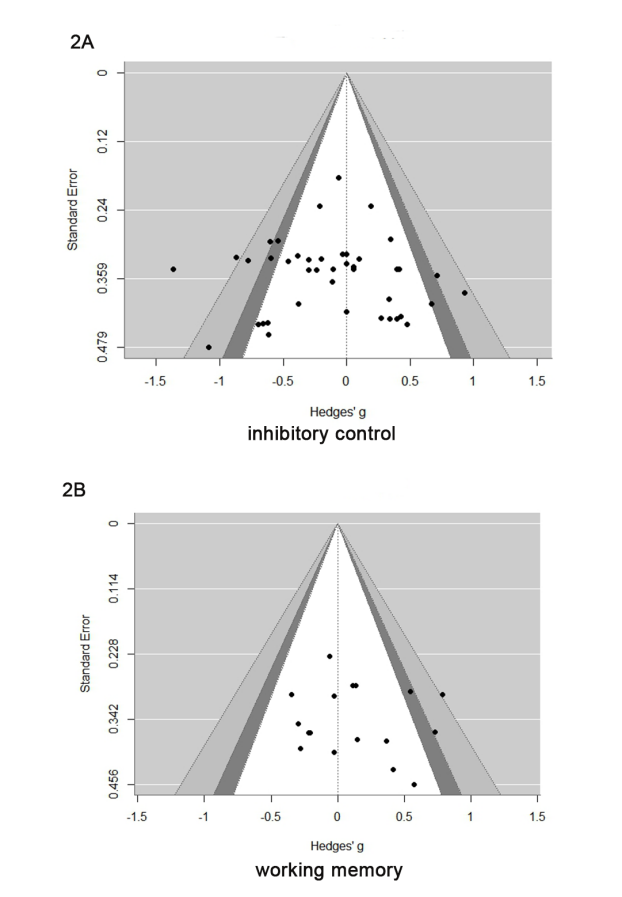


**Supplementary Figure S2**: Funnel plots for included studies on inhibitory control functions and working memory.

**SupplementaryTableS1:Summaryof Findings (SoF) Table**

| **Certainty assessment** | | | | | | | **№ of patients** | | **Effect** | | **Certainty** | **Importance** |
| --- | --- | --- | --- | --- | --- | --- | --- | --- | --- | --- | --- | --- |
| **№ of studies** | **Study design** | **Risk of bias** | **Inconsistency** | **Indirectness** | **Imprecision** | **Other considerations** | **Transcranial Direct Current Stimulation** | **Sham** | **Relative (95% CI)** | **Absolute (95% CI)** |  |  |
| **Inhibition Control** | | | | | | | | | | | | |
| 20 | randomised trials | serious^a^ | not serious | not serious | not serious | none | 431 | 441 | - | SMD **0.1 SD lower** (0.26 lower to 0.05 lower) | ⨁⨁⨁◯ Moderate^a^ | CRITICAL |
| **Working Memory** | | | | | | | | | | | | |
| 12 | randomised trials | serious^a^ | not serious | not serious | not serious | none | 250 | 256 | - | SMD **0.13 SD higher** (0.06 lower to 0.32 higher) | ⨁⨁⨁◯ Moderate^a^ | CRITICAL |
| **Hot Executive Function** | | | | | | | | | | | | |
| 2 | randomised trials | serious^a^ | not serious | not serious | not serious | publication bias strongly suspected^b^ | 43 | 43 | - | SMD **0.27 SD higher** (0.14 lower to 0.70 higher) | ⨁⨁◯◯ Low^a,b^ | IMPORTANT |
| **Cognitive Flexibility** | | | | | | | | | | | | |
| 3 | randomised trials | serious^a^ | not serious | not serious | not serious | publication bias strongly suspected^a,b^ | 60 | 62 | - | SMD **0.05 SD higher** (0.49 lower to 0.58 higher) | ⨁⨁◯◯ Low^a,b^ | IMPORTANT |
| **Adverse Reactions-SKIN** | | | | | | | | | | | | |
| 5 | randomised trials | serious^a^ | not serious | not serious | not serious | none | 194/516 (37.6%) | 141/556 (25.4%) | **RR 1.39** (1.18 to 1.63) | **99 more per 1,000** (from 46 more to 160 more) | ⨁⨁⨁◯ Moderate^a^ | IMPORTANT |

**CI:** confidence interval; **RR:** risk ratio; **SMD:** standardised mean difference

#### Explanations：a. The number of studies was small. Some studies only blinded participants but not staff, and the description of allocation concealment was inadequate in some studies.b. The number of studies was very limited.

**Author(s):** Jie li,Xinyu Hou **Question:** Transcranial Direct Current Stimulation compared to Sham in ADHD

**Setting:** effective and safety

| **Supplementary TableS2. Subgroup and Meta-Regression Analyses Results of Inhibition Control** | | | | |
| --- | --- | --- | --- | --- |
| **Analysis Category** | **Subgroup / Variable** | **Estimate (95% CI)** | **p-value (Interaction)** | **p-value**  **(Within Subgroup)** |
| **Task Type​** | **CPT** | **0.09 (-0.28, 0.45)** | **0.55** | **0.64** |
|  | **Flanker** | **-0.20 (-0.53, 0.14)** |  | **0.25** |
|  | **No-Go** | **-0.17 (-0.40, 0.07)** |  | **0.16** |
|  | **Other** | **-0.17 (-0.63, 0.28)** |  | **0.46** |
|  | **Stroop** | **0.05(-0.39, 0.49)** |  | **0.82** |
| **Electrode Position​** | **F3** | **-0.04 (-0.26, 0.19)** | **0.19** | **0.75** |
|  | **F4** | **0.03 (-0.26, 0.31)** |  | **0.87** |
|  | **F8** | **-0.22 (-0.61, 0.18)** |  | **0.29** |
|  | **Fp2** | **-0.52 (-0.94, -0.11)** |  | **0.01** |
| **Design​** | **Parallel** | **0.04 (-0.24, 0.33)** | **0.16** | **0.76** |
|  | **Crossover** | **-0.17 (-0.35, 0.01)** |  | **0.06** |
| **Brain Region​** | **DLPFC** | **-0.08 (-0.66, 0.51)** | **0.60** | **0.80** |
|  | **L-DLPFC** | **-0.13 (-0.34, 0.08)** |  | **0.24** |
|  | **R-DLPFC** | **0.04 (-0.30, 0.38)** |  | **0.82** |
|  | **R-IFG** | **-0.31 (-0.70, 0.07)** |  | **0.11** |
|  | **r-PPC** | **0.23 (-0.48, 0.94)** |  | **0.53** |
| **Meta-Regression​** | **Number of Sessions​** | **0.03 (-0.003, 0.07)** | **0.07** | **0.07** |
|  | **Stimulation Intensity​** | **-0.17 (-0.51, 0.17)** | **0.32** | **0.32** |
| **Age Group​** | **Adult** | **-0.24 (-0.53, 0.06)** | **0.25** | **0.12** |
|  | **Children** | **-0.06 (-0.24, 0.13)** |  | **0.56** |
| **Stimulation Duration​** | **≤15 min** | **-0.20 (-0.56, 0.17)** | **0.52** | **0.25** |
|  | **16-20 min** | **-0.05(-0.62, 0.52)** |  | **0.85** |
|  | **26-30 min** | **-0.22 (-0.67, 0.23)** |  | **0.33** |

| **Supplementary TableS3. Subgroup and Meta-Regression Analyses Results of Working Memory** | | | | |
| --- | --- | --- | --- | --- |
| **Analysis Category** | **Subgroup / Variable** | **Estimate**  **(95% CI)** | **p-value (Interaction)** | **p-value (Within Subgroup)** |
| **Age Group​** | **Adult** | **0.08 (-0.42, 0.59)** | **0.44** | **0.74** |
|  | **Children** | **0.14 (-0.08, 0.36)** |  | **0.22** |
| **Meta-Regression​** | **Stimulation Intensity​** | **0.14 (-0.28, 0.55)** | **0.52** | **0.52** |
|  | **Number of Sessions​** | **-0.009 (-0.05, 0.03)** | **0.65** | **0.65** |
| **Brain Region​** | **L-DLPFC** | **-0.07 (-0.59, 0.45)** | **0.69** | **0.79** |
|  | **L-DLPFC** | **0.04 (-0.34, 0.42)** |  | **0.83** |
|  | **R-DLPFC** | **0.30 (-0.22, 0.82)** |  | **0.26** |
|  | **R-IFG** | **0.19 (-0.22, 0.60)** |  | **0.37** |
| **Electrode Position​** | **F3** | **-0.008 (-0.24, 0.23** | **0.26** | **0.95** |
|  | **F4** | **0.24 (-0.17, 0.66)** |  | **0.25** |
|  | **F8** | **0.13 (-0.19, 0.45)** |  | **0.42** |
|  | **Fp2** | **0.72 (0.004, 1.44)** |  | **0.044​** |
| **Task Type​** | **N-back** | **0.15 (-0.04, 0.35)** | **0.04** | **0.13** |
|  | **Other** | **-0.15 (-0.64, 0.34)** |  | **0.55** |
| **Stimulation Duration (Group)​** | **≤15 min** | **0.26 (-0.33, 0.85)** | **0.45** | **0.39** |
|  | **16-20 min** | **0.03 (-0.20, 0.26)** |  | **0.81** |
|  | **26-30 min** | **0.28 (-0.13， 0.69,)** |  | **0.17** |

| **Supplementary TableS4. Subgroup and Meta-Regression Analyses Results of Hot Executive Function** | | | | | | |
| --- | --- | --- | --- | --- | --- | --- |
| **Analysis Category** | **Subgroup / Variable** | **Estimate (95% CI)** | **p-value (Interaction)** | **p-value (Within Subgroup)** | | |
| **Brain Region​** | **DLPFC** | **0.40 (-0.21, 1.02)** | **0.39** | | **0.20** |  |
|  | **vmPFC** | **0.16 (-0.46, 0.77)** |  | | **0.62** |  |
| **Electrode Position​** | **F3** | **0.40 (-0.21, 1.02)** | **0.39** | | **0.20** |  |
|  | **Fp2** | **0.16 (-0.46, 0.77)** |  | | **0.62** |  |
| **Meta-Regression​** | **Number of Sessions​** | **0.16 (-0.50, 0.82)** | **0.64** | | **0.64** |  |
|  | **Stimulation Intensity​** | **0.64 (-2.0, 3.27)** | **0.64** | | **0.64** |  |

| **Table S5:Results of sensitivity analysis of Inhibition Control** | | | | |
| --- | --- | --- | --- | --- |
| **Excluded_study** | **Estimate** | **Standard Error** | **95%CI** | **p_value** |
| **Allenby_2018** | **-0.11** | **0.09** | **[0.28,0.06]** | **0.20** |
| **Barham_2022** | **-0.10** | **0.08** | **[0.26,0.07]** | **0.26** |
| **Beiman_2025** | **-0.11** | **0.08** | **[0.27,0.05]** | **0.19** |
| **Breitling_2016** | **-0.07** | **0.06** | **[0.20,0.05]** | **0.25** |
| **BreitlingZiegler_2021** | **-0.12** | **0.08** | **[0.28,0.04]** | **0.14** |
| **Cosmo_2015** | **-0.11** | **0.08** | **[0.27,0.06]** | **0.21** |
| **DubreuilVall_2021** | **-0.10** | **0.09** | **[0.28,0.07]** | **0.25** |
| **Estaji_2024** | **0.08** | **0.08** | **[0.24,0.08]** | **0.31** |
| **Jacoby and Lavidor_2018** | **-0.11** | **0.08** | **[0.27,0.06]** | **0.20** |
| **Klomjai_2022** | **-0.11** | **0.08** | **[0.27,0.05]** | **0.16** |
| **Krauel_2025** | **-0.12** | **0.09** | **[0.29,0.04]** | **0.15** |
| **Nejati_2020** | **-0.08** | **0.09** | **[0.26,0.09]** | **0.34** |
| **Nejati_2021** | **-0.10** | **0.08** | **[0.27,0.06]** | **0.23** |
| **Nejati_2022** | **-0.12** | **0.08** | **[0.28,0.04]** | **0.13** |
| **Salehinejad_2020** | **-0.12** | **0.08** | **[0.28,0.04]** | **0.15** |
| **Salehinejad_2022** | **-0.10** | **0.08** | **[0.26,0.07]** | **0.24** |
| **Schertz_2022** | **-0.10** | **0.08** | **[0.26,0.07]** | **0.24** |
| **Sotnikova A_2017** | **-0.12** | **0.08** | **[0.28,0.04]** | **0.15** |
| **Westwood_2021** | **-0.12** | **0.08** | **[0.28,0.04]** | **0.14** |
